# Expression Profiling of Adipogenic and Anti-Adipogenic MicroRNA Sequences following Methylmercury Exposure in *Caenorhabditis elegans*

**DOI:** 10.3390/toxics11110934

**Published:** 2023-11-17

**Authors:** Giancarlo Garofalo, Tyson Nielsen, Samuel Caito

**Affiliations:** Department of Pharmaceutical Sciences, Husson University School of Pharmacy, Bangor, ME 04401, USA

**Keywords:** methylmercury, lipid homeostasis, miRNA

## Abstract

MicroRNA (miRNA) are important regulators of gene expression that respond not only to developmental and pathological cues, but also to environmental stimuli. Dyslipidemia is a hallmark of metabolic conditions and has been shown to significantly affect the expression of circulating miRNA sequences. Recently, our lab has shown that the environmental toxicant methylmercury (MeHg) causes dyslipidemia in the *Caenorhabditis elegans* model organism. While 10 and 20 μM MeHg increases the expression of adipogenic transcription factors and lipid-binding proteins in worms, there is limited information on how the toxicant affects the miRNA regulators of these genes. We hypothesized that MeHg would increase the expression of adipogenic miRNA sequences and/or decrease the expression of anti-adipogenic miRNA sequences. We further hypothesized that the target mRNA sequences for the miRNAs affected by MeHg would be consequently altered. We selected three potentially adipogenic (*mir-34*, *mir-124*, and *mir-355*) and three potentially anti-adipogenic *(mir-240*, *mir-786*, and *let-7*) miRNA sequences homologous to known human miRNA sequences altered in obesity, and quantified their levels 24 h and 48 h post MeHg treatment. At 24 h post exposure, MeHg significantly increased expression of both the adipogenic and anti-adipogenic miRNA sequences 1.5–3x above untreated control. By 48 h post exposure, only the adipogenic miRNA sequences were elevated, while the anti-adipogenic miRNA sequences were decreased by 50% compared to untreated control. These data suggest that there are developmental changes in miRNA expression over time following MeHg exposure. We next selected one target mRNA sequence for each miRNA sequence based on miRNA–mRNA relationships observed in humans. MeHg altered the gene expression of all the target genes assayed. Except for *mir-34*, all the tested miRNA–mRNA sequences showed a conserved relationship between nematode and humans. To determine whether the selected miRNA sequences were involved in lipid accumulation in response to MeHg, lipid storage was investigated in transgenic worm strains that lacked the specific miRNA strains. Of the six strains investigated, only the *mir-124* and *let-7* mutant worms had lipid storage levels that were statistically different from wild type, suggesting that these two sequences can be potential mediators of MeHg-induced lipid dysregulation.

## 1. Introduction

Dyslipidemia is a condition where serum lipid levels of cholesterol, low-density lipoprotein (LDL), high-density lipoprotein (HDL), and triglycerides become imbalanced. Imbalance of serum lipids can occur due to a variety of means, such as over production of cholesterol, elevated levels of LDL or decreased levels of HDL. Dyslipidemia can arise through a variety of sources, including genetics, diet, or other environmental factors, and can predispose individuals to cardiovascular disease and metabolic syndrome (MS). MS is defined as a multifactorial condition characterized by obesity, insulin resistance, diabetes mellitus (DM), and dyslipidemia. Environmental factors have recently been implicated in the development of MS [1,2].

Methylmercury (MeHg) is a well-known neurotoxin that also has metabolic effects. Inorganic Hg is released through volcanic eruption as well as by human-driven processes, such as mining, industrial production and waste, as well as artisanal gold processing [3]. This inorganic Hg is biomethylated to MeHg by microbes. MeHg bioaccumulates and biomagnifies up the food chain, where humans are exposed to it via fish and seafood consumption. The highest concentrations of MeHg are present in large predatory species, such as tuna, mackerel, swordfish, and sharks [3]. Significant levels of MeHg have also been found in rice and poultry in China [4,5]. Developmental exposure to MeHg causes behavioral and cognitive dysfunction in children. Cumulative exposure to MeHg over an adult’s lifetime has been linked to movement disorders and the development of neurodegenerative diseases, such as Parkinson’s disease [3,6]. Increased blood and toenail mercury levels in individuals with high fish consumption have been associated with the development of MS, obesity, and lipid dysregulation [7,8,9], as well as increased visceral adipose tissue [10,11]. Furthermore, exposure to environmentally relevant concentrations of MeHg has been shown to increase lipid dysregulation and aggravate cardiovascular risk factors in wild-type and in ApoE knockout mice [12,13]. Elevations in cholesterol and triglycerides in response to MeHg have been observed in experimental animals as diverse as rodents, fish, and worms [14,15,16]. We have previously demonstrated that acute early-life exposure to 10 and 20 μM MeHg causes lipid dysregulation in *Caenorhabditis elegans* (*C. elegans*) by increasing the size and number of lipid storage sites, triglyceride levels, and altering gene expression for genes associated with lipid synthesis, transport, and storage [17]. The metabolic effects of MeHg in *C. elegans* were also shown to be modified by diet; the lower the lipid content of the diet, the less lipid dysregulation was observed in worms exposed to MeHg [18]. Additionally, lipid dysregulation could be modified by altering the ability of the worms to produce miRNAs. Worms that expressed more miRNA than wild type were protected from MeHg toxicity and lipid dysregulation, whereas worms that could not produce functional miRNA had exacerbated toxicity and lipid dysregulation in response to MeHg [19].

MiRNAs are short, non-coding RNAs comprised of 20–24 nucleotides in length. These RNA molecules work post-transcriptionally to repress the expression of target genes by base pairing with the 3′ UTR region of mRNA target sequences, leading to translational repression [20]. MiRNA are responsive to environmental, pharmacological, and pathological stimuli, and thereby are useful as biomarkers of disease and chemical exposure in humans [20]. MiRNA expression has been shown to be altered in serum and adipose tissue of patients with obesity, type 2 diabetes, and heart disease [20,21,22,23,24,25,26,27]. Some miRNAs act to increase lipid content of adipocytes or serum and are found in higher levels in serum of obese individuals than in non-obese individuals [21,22,23,24,25,26]. Other miRNA sequences are expressed highly in non-obese individuals but are lower in expression in obese individuals [21,22,23,24,25,26]. These miRNA sequences repress lipid accumulation pathways or increase lipid oxidation in organs. Characterization of the adipogenic potential of individual miRNA sequences has mainly been conducted in mammalian adipocyte cell culture, human patients, and rodents.

*C. elegans* can be a useful tool in screening miRNA sequences for their potential to cause lipid dysregulation in response to environmental factors, such as MeHg. *C. elegans* contains homologs for many of the miRNA that mammals express [27], and some miRNA that mammals express were first identified in *C. elegans*, such as *let-7*. *C. elegans’ let-7* is a multifunctional miRNA that is highly conserved (80–100%) with the human let-7 family (let-7 a-k) [28,29]. In 3T3-L1 adipocytes, *let-7* has been shown to inhibit adipogenesis mediated by PPARγ [30]. *Let-7* is also a regulator of insulin resistance; *let-7* knockout mice do not develop insulin resistance despite diet-induced obesity [31]. In *C. elegans*, *let-7* has been shown to regulate lipid levels by modulating the mTORC2/PQM-1 signaling pathway in the intestine [32]. However, for many of the miRNA sequences with high sequence homology to human miRNA sequences, conservation of mRNA targets or physiological effects due to changes in their expression have not been characterized. Worms contain homologous machinery for the expression of miRNA as well as for lipid metabolic pathways. The ease of genetic manipulation allows for the generation of miRNA-deficient worm strains, which can be used to screen for physiological effects of MeHg or other environmental contaminants.

Recently we have described how miRNA expression can affect how *C. elegans* respond to MeHg. Worms that could not express functional miRNA were more sensitive to MeHg, had increased oxidative burden, triglyceride content, and lipid storage sites, as well as increased feeding behaviors [19]. These data suggested that MeHg exposure induced expression of miRNA that could modify these responses in wild-type worms but were not present in the miRNA-deficient strains. We therefore hypothesized that MeHg would increase the expression of the potentially adipogenic miRNA sequences and/or decrease the expression of the potentially anti-adipogenic miRNA sequences. We tested this hypothesis by quantifying three miRNA sequences shown in mammals to be adipogenic and three miRNA sequences shown in mammals to be anti-adipogenic at various time points following MeHg exposure in *C. elegans*. We assessed whether altered miRNA expression in response to MeHg leads to altered gene expression of known mRNA sequences for each miRNA. Finally, we assessed whether expression of any of the six miRNA sequences affected lipid accumulation in *C. elegans* basally and in response to MeHg.

## 2. Materials and Methods

### 2.1. Reagents

All reagents were obtained from Sigma-Aldrich (St. Louis, MO, USA), unless otherwise stated. Methylmercury (II) chloride, >99.5% purity (Sigma, catalog number 442534). Primers used in this study include *aak-2* (F: TTGCTCACGAGTGGTTCCAG, R: CGCTGAAACTCAGTGACCTCT), *atgl-1* (F: CCGACTACAAGTAAACGTGATGC, R: GGTGGCTGGGATGATGTGAA), *daf-2* (F: GCTCTCGGAACAACCACTGA, R: TGAATCGGGCGTCGTTCTAC), *lbp-5* (F: CGTGTGCTGCAAAACCAACT, R: CTCGTCGAATTCCACTCCCA), *paqr-2* (F: TTCGTTGGCACTTTGCTTGG, R: ATAAACCAACATCCGCCGGT), and *tba-1* (F: AGACCAACAAGCCGATGGAG, R: TCCAGTGCGGATCTCATCAAC). TaqMan^TM^ miRNA expression assays used in this study include *mir-34-3p* (241995_mat), *mir-124-5p* (243964_mat), *mir-240-3p* (001373_mat), *mir-355*-*5p* (244849_mat), *mir-786-3p* (461852_mat), *let-7a-5p* (4427975_mat), and U18 (001764).

### 2.2. C. elegans Strains and Handling of the Worms

*C. elegans* strains were maintained at 25 °C on Nematode Growth Medium (NGM) agar plates seeded with the OP-50 strain of *Escherichia coli*, as previously described [33]. The following strains were used in this study: wild-type N2, MT7626 (*let-7*(n2853) X), MT13406 (*mir-34*(n4276) X), MT15873 (*mir-240*(n4541) X), MT16316 (*mir-355*(n4618) II), MT18043 (*mir-240* and *mir-786*(n4541) X), and VT2527 (*mir*-124(n4255) IV). All strains were obtained from the *Caenorhabditis* Genetic Center (CGC; University of Minnesota). Populations of worms were synchronized to the same developmental stage (L1) using a bleaching method [34]. Eggs were isolated from gravid worm populations using a bleach solution (1% NaOCl and 0.25 M NaOH) followed by a 30% sucrose gradient to separate eggs from worm and bacterial debris. L1 worms were then treated with various MeHg concentrations for 30 min in liquid M9 buffer at 25 °C on an Eppendorf tube rotator. For N2 worms, we have previously shown 1, 10, and 20 μM are below the lethal dose 50% (LD_50_) for MeHg in *C. elegans* and correlate to intracellular concentrations of MeHg that are below the US EPA reference dose of 0.1 μg/kg/d [35,36]. For experiments utilizing the transgenic miRNA strains, concentrations of MeHg were chosen below the LD_50_ for each individual strain.

### 2.3. RNA Isolation, miRNA Expression, and Real-Time qPCR Gene Expression

Twenty thousand worms were treated with MeHg, and RNA was isolated 24 or 48 h later using Trizol followed by chloroform extraction, as previously described [37]. After RNA extraction, miRNA was synthesized from 1 mg of total RNA using the TaqMan™ MicroRNA Reverse Transcription Kit (Thermo Fisher Scientific, Waltham, MA, USA). Specific TaqMan^®^ miRNA expression assays for the miRNA sequences were used according to manufacturer’s instructions. Alternatively, following RNA extraction, cDNA was synthesized from 1 mg of total RNA using the Applied Biosystems’ High-Capacity cDNA Reverse Transcription kit (Thermo Fisher Scientific). Real-time PCR analysis was performed using PerfeCTa SYBR Green FastMix (QuantaBio, Beverly, MA, USA). The housekeeping genes used to normalize miRNA data were U18 and *tba-1* for mRNA.

### 2.4. Dose–Response Curves

The lethal dose (LD_50_) of MeHg for the six mutant *C. elegans* strains was determined by treating 5000 synchronized L1 worms with doses ranging from 0 to 200 μM MeHg. All exposures were performed in triplicate and repeated 5 times. After treatment, worms were washed 3 times with M9 buffer, then transferred to OP-50-seeded NGM plates, and manually counted for lethality after 24 h.

### 2.5. Nile Red Staining

We have previously shown that fat storage sites are increased by 10 and 20 μM MeHg in N2 worms as assessed by three different dyes: BODIPY 493/503, Oil Red O, and Nile Red [17]. Of the three methods, Nile Red is optimal for screening multiple genotypes and treatment groups. Following MeHg treatment, 20,000 L1 worms were washed with M9 and transferred to agar plates for 72 h. The worms were then washed off the agar plates and manually counted, placing 1000 worms per treatment in Eppendorf tubes for fixation. Fixation and Nile Red staining was performed as previously described [38]. Worms were first washed with 0.1% Triton X-100 in PBS, fixed in 40% isopropanol for 3 min, and then incubated with 3 μg/mL Nile Red in 40% isopropanol for 30 min. After staining, worms were washed with M9 and loaded onto a 96-well black microtiter plate. Nile Red fluorescence was read at excitation 560 nm and emission 590 nm. Data were normalized to worm number and protein levels.

### 2.6. Statistics

Statistical analyses were performed using Prism 8.4.3 software (Graphpad, San Diego, CA, USA). Data were tested for normal distribution using the Shapiro–Wilk test, all of which had *p* values indicating normality. Statistical analysis of significance was carried out either using Student’s *t*-test for LD_50_ value comparisons or two-way analysis of variance (ANOVA) for all other data. Values of *p* < 0.05 were considered statistically significant.

## 3. Results

### 3.1. MeHg Increases Adipogenic miRNA Expression and Decreases Anti-Adipogenic miRNA Expression

**Figure 1:**
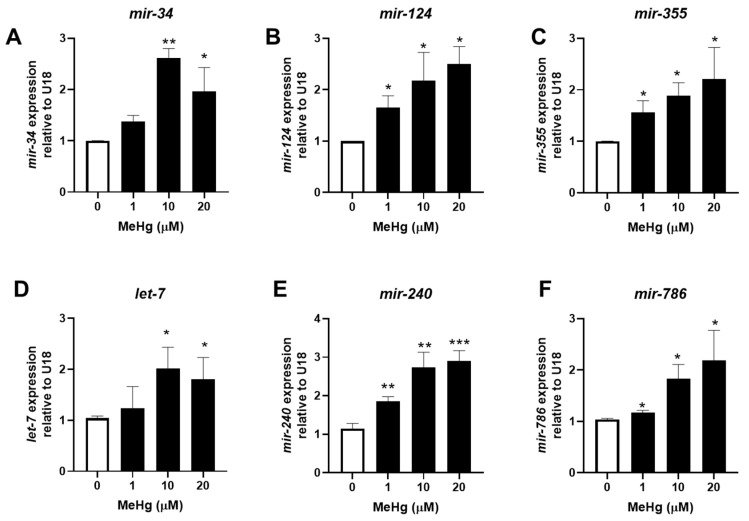
Methylmercury increases miRNA expression 24 h post exposure. N2 worms were exposed to either 0, 1, 10, or 20 μM of MeHg for 30 min and harvested 24 h later. Levels of adipogenic miRNA sequences (**A**) *mir-34*, (**B**) *mir-124*, (**C**) *mir-355* and anti-adipogenic miRNA sequences (**D**) *let-7*, (**E**) *mir-240*, and (**F**) *mir-786* were quantified using qPCR and normalized to U18 C/D box small nucleolar RNA as housekeeping gene. Data are representative of the mean ± SEM from 6 independent experiments. * *p*  <  0.05, ** *p* < 0.01, *** *p* < 0.001 as compared with untreated control.

**Figure 2:**
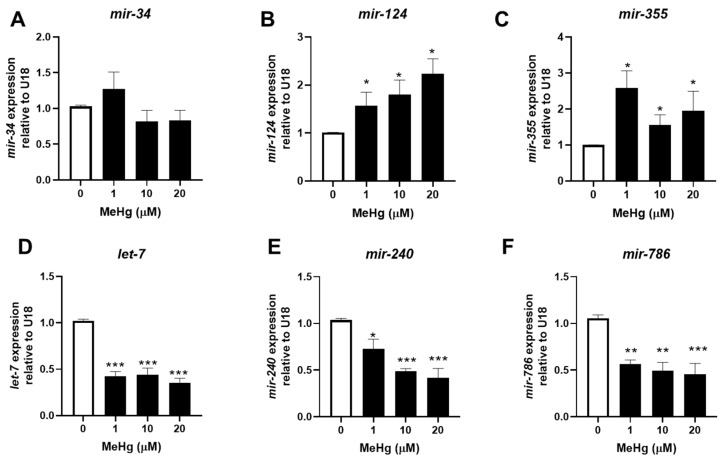
Methylmercury increases adipogenic miRNA expression and decreases anti-adipogenic miRNA expression 48 h post exposure. N2 worms were exposed to either 0, 1, 10, or 20 μM of MeHg for 30 min and harvested 48 h later. Levels of adipogenic miRNA sequences (**A**) *mir-34*, (**B**) *mir-124*, (**C**) *mir-355* and anti-adipogenic miRNA sequences (**D**) *let-7*, (**E**) *mir-240*, and (**F**) *mir-786* were quantified using qPCR and normalized to U18 C/D box small nucleolar RNA as housekeeping gene. Data are representative of the mean ± SEM from 6 independent experiments. * *p*  <  0.05, ** *p* < 0.01, *** *p* < 0.001 as compared with untreated control.

We have previously shown that genetically decreasing the expression of total miRNA significantly increased the toxicity of MeHg in *C. elegans* [19]. Therefore, we were interested in how individual miRNA sequences might change following MeHg exposure. We selected three nematode miRNA sequences with high homology to mammalian miRNA that have been shown to be increased and three sequences that have been shown to be decreased in the serum of obese individuals (Table 1). RNA was collected from MeHg-treated wild-type (N2) worms 24 h and 48 h post exposure, and miRNA expression was then quantified. After 24 h, MeHg significantly increased the levels of all six sequences above the level of untreated control (Figure 1). By 48 h post exposure, the adipogenic sequences *mir-124* and *mir-355* remained elevated by MeHg, while the anti-adipogenic miRNA sequences were decreased below the level of the untreated control (Figure 2). The adipogeinc *mir-34* levels were elevated by MeHg exposure at 24 h, but were not significantly different from the untreated worms at 48 h. These data suggest that MeHg increases the expression of adipogenic *mir-124* and *mir-355* sequences for several hours post treatment in worms. Conversely, MeHg initially increased production of the anti-adipogenic *let-7*, *mir-240*, and mir-786 sequences, but the levels dropped over time post treatment. Both the increased adipogenic miRNA sequence levels and decreased anti-adipogenic miRNA sequence levels could lead to a gene expression profile that promotes dyslipidemia in response to MeHg.

### 3.2. MeHg Decreases the Expression of Anti-Adipogenic Genes Modulated by mir-124 and mir-355

**Figure 3:**
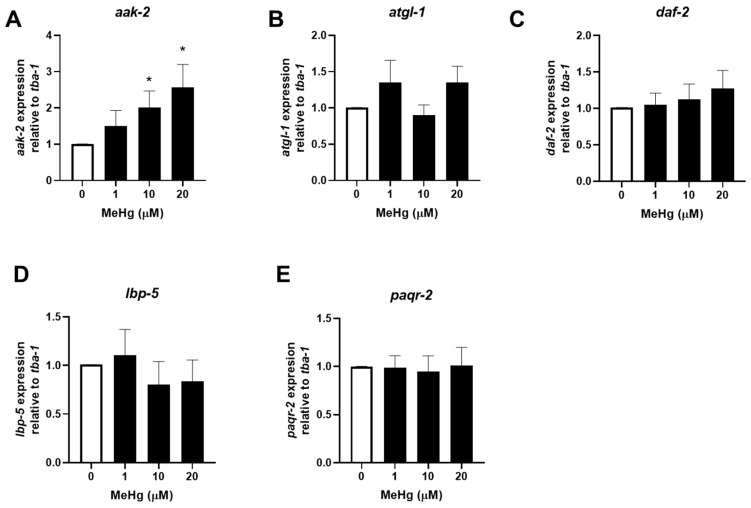
MeHg alters *aak-2* but none of the other mRNA sequences of miRNA-responsive genes 24 h post exposure. N2 worms were exposed to either 0, 1, 10, or 20 μM of MeHg for 30 min and harvested 24 h later. Total RNA was extracted, cDNA was made, and used for qPCR. Expression of (**A**) *aak-2*, (**B**) *atgl-1*, (**C**) *daf-2*, (**D**) *lbp-5*, and (**E**) *paqr-2* were compared relative to tubulin alpha chain 1 (*tba-1*). Data are representative of the mean ± SEM from 6 independent experiments. * *p*  <  0.05 as compared with untreated control.

**Figure 4:**
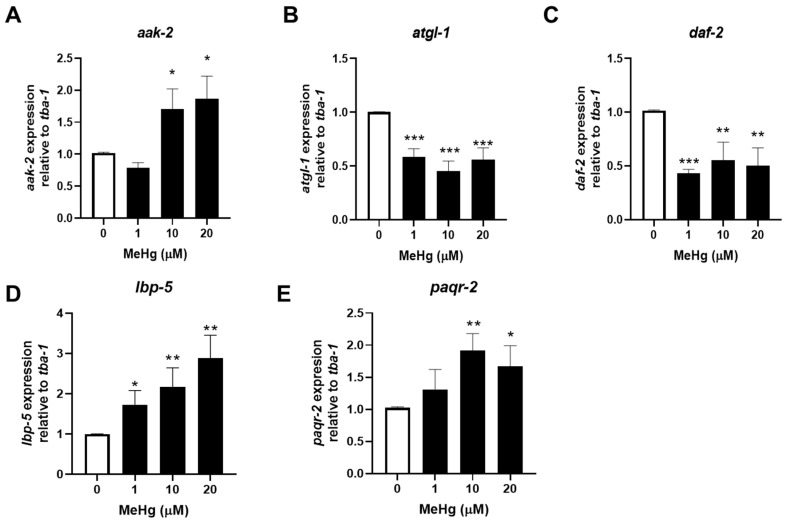
MeHg alters mRNA expression of miRNA-responsive genes 48 h post exposure. N2 worms were exposed to either 0, 1, 10, or 20 μM of MeHg for 30 min and harvested 48 h later. Total RNA was extracted, cDNA was made, and used for qPCR. Expression of (**A**) *aak-2*, (**B**) *atgl-1*, (**C**) *daf-2* (**D**) *lbp-5* and (**E**) *paqr-2* were compared relative to tubulin alpha chain 1 (*tba-1*). Data are representative of the mean ± SEM from 6 independent experiments. * *p*  <  0.05, ** *p* < 0.01, *** *p* < 0.001 as compared with untreated control.

To examine whether the increased levels of *mir-34*, *mir-124*, and *mir-355* in response to MeHg would alter gene expression, we quantified gene expression of one target mRNA for each miRNA (Table 1). We selected the mRNA target genes based on the criteria that the gene has been shown to be regulated by miRNA in mammals and that there exists a well-characterized orthologous gene in nematodes. As MeHg increases the expression of *mir-124* and *mir-355*, we hypothesized that the target sequences would decrease in expression in response to MeHg treatment. RNA was collected 24 and 48 h post MeHg exposure and target gene expression was quantified using real-time qPCR. At 24 h, *aak-2*, the target gene for *mir-34*, was increased, while *atgl-1* and *daf-2* (target genes for *mir-124* and *mir-355*, respectively) showed no significant change in expression in response to MeHg as compared to untreated control (Figure 3A–C). In Figure 1, we show that *mir-34* levels in response to MeHg were increased at 24 h; we therefore expected that *aak-2* levels would decrease. However, MeHg increased *aak-2* levels at 24 h (Figure 3A). Similarly at 48 h, MeHg increased the expression of *aak-2*, (Figure 4A) while *mir-34* levels were unchanged by MeHg (Figure 2). Since *aak-2* was actually increased by MeHg at both 24 and 48 h independent of *mir-34* levels, our data suggest that either *mir-34* does not regulate the *aak-2* sequence in nematodes or that there are other regulators of *aak-2* that are altered by MeHg. This suggests that other gene expression regulators are activated by MeHg that lead to an increase in *aak-2* expression. At 48 h, *atgl-1* and *daf-2* were both decreased in response to MeHg (Figure 4B,C). The increased *mir-124* and *mir-355* expression and decreased *atgl-1* and *daf-2* expression confirmed the miRNA–mRNA relationship in nematodes and humans. Furthermore, it demonstrates how MeHg-induced changes in miRNA have consequences on mRNA expression.

### 3.3. MeHg Increases the Expression of Adipogenic Genes Modulated by let-7, mir-240, and mir-786

To examine whether the decreased expression levels of *let-7*, *mir-240*, and *mir-786* in response to MeHg would alter gene expression, we selected one gene that has been shown, in humans, to be a target for each miRNA sequence (Table 1). As MeHg decreases the expression of *let-7*, *mir-240, and mir-786*, we hypothesized that the target sequences would increase in expression in response to MeHg treatment. RNA was collected 24 and 48 h post MeHg exposure and target gene expression was quantified using real-time qPCR. At 24 h, the expression of *lbp-5* (target mRNA for *let-7*) and *paqr-2* (target mRNA for both *mir-786* and *mir-240*) levels were unchanged in response to MeHg treatment (Figure 3D,E). However, at 48 h, MeHg increased the expression of both *lbp-5* and *paqr-2* (Figure 4D,E). The decreased *let-7*, *mir-240*, and *mir-786* expression and increased *lbp-5* and *paqr-2* expression confirmed the miRNA–mRNA relationship in nematodes and humans.

### 3.4. Reduced miRNA Expression Sensitizes Worms to MeHg Toxicity

**Figure 5:**
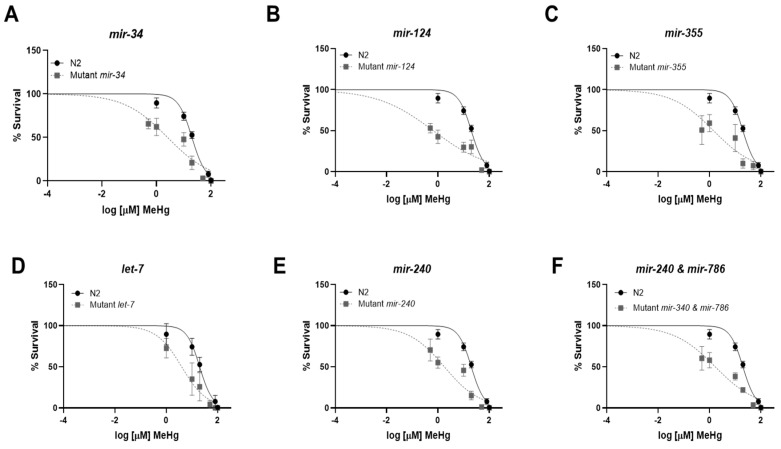
Knockout of specific miRNA sequences affects MeHg toxicity. N2 and transgenic worms with mutations in (**A**) *mir-134*, (**B**) *mir-124*, (**C**) *mir-355*, (**D**) *let-7*, (**E**) *mir-240*, and (**F**) *mir-240* and *mir-786* were treated with increasing concentrations of MeHg for 30 min and transferred to agar plates spread with OP50 *E. coli*. Worms were manually counted 24 h post exposure. Dose–response curves were generated from five independent experiments.

We have previously shown that worms with altered miRNA expression capabilities have differential sensitivity to MeHg as compared to wild-type worms [19]. To examine the effect of deleting specific miRNA sequences on the worm’s sensitivity to MeHg, we performed a survival dose–response assay. We exposed wild-type N2 worms, mutant *mir-34*, *mir-124*, *mir-355*, *mir-240*, *mir-240 and mir-786*, and *let-7* strains to increasing concentrations of MeHg, and generated dose–response survival curves (Figure 5). Table 2 presents the LD_50_ concentrations of MeHg for each strain. All mutant miRNA strains of worms showed significant left-hand shifts in the survival curves as compared to N2, indicating that deletion of the specific miRNA strain sensitizes worms to the toxic effects of MeHg. These data suggest that each of these miRNA may have additional roles in either development, xenobiotic metabolism, or stress responses that when lost, make the worms more susceptible to exposure to MeHg.

### 3.5. MiRNA Expression Influences MeHg-Induced Lipid Accumulation

**Figure 6:**
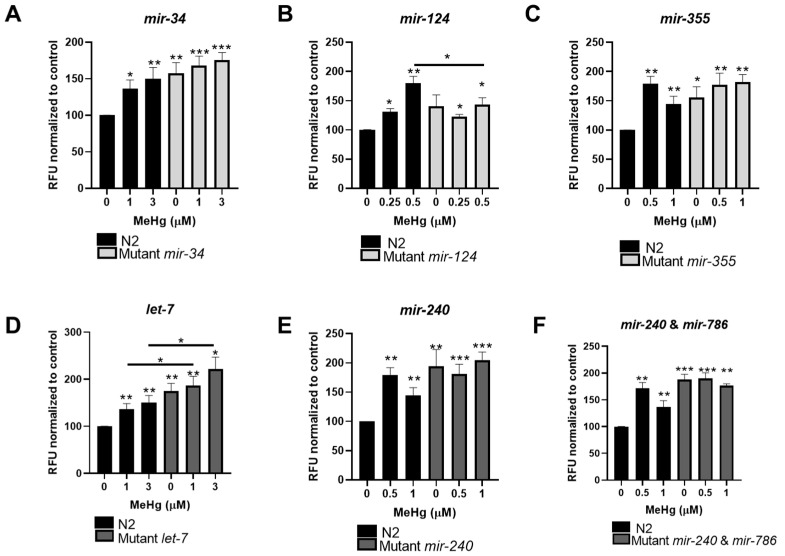
Lipid content in response to MeHg is affected by miRNA expression. Lipid storage sites were quantified from N2 and transgenic worms with mutations in (**A**) *mir-134*, (**B**) *mir-124*, (**C**) *mir-355*, (**D**) *let-7*, (**E**) *mir-240*, and (**F**) *mir-240* and *mir-786* treated with MeHg for 30 min. At 72 h after MeHg treatment, worms were fixed, stained with Nile Red, and fluorescence was measured. Data are expressed as mean Nile Red fluorescence normalized to worm number and protein content  ± SEM from five independent experiments. * *p*  <  0.05, ** *p* < 0.01, *** *p* < 0.001 as compared with untreated N2 control.

Lastly, we examined whether deleting any of these miRNA sequences could affect lipid accumulation in response to MeHg. It was hypothesized that the deletion of either of the adipogenic miRNAs would prevent lipid accumulation in response to MeHg, while deletion of either of the anti-adipogenic miRNAs would augment lipid accumulation in response to MeHg. N2 and mutant L1 worms were treated with MeHg and lipid storage sites were stained with Nile Red in adult worms (72 h after exposure). As the mutant worms were more susceptible to MeHg than N2, MeHg concentrations were selected at each strain’s LD_50_ or at each strain’s LD_25_. Interestingly, treatment of N2 worms with MeHg increased lipid accumulation at all concentrations (0.25, 0.5, 1, and 3 μM) (Figure 6A–F).

In examining lipid storage in the deletion mutants for the adipogenic miRNA sequence strains, we expected the untreated mutants to have similar or lower lipid storage levels to the N2 untreated worms. Surprisingly, the untreated mutant *mir-34* and mutant *mir-355* worms had significantly more lipid storage than the untreated N2 worms (Figure 6A,C). Furthermore, while both mutant *mir-34* and mutant *mir-355* worms treated with MeHg had elevated lipid storage, there was no statistical difference between the MeHg-treated N2 worms and the MeHg-treated mutant worms. There was also no difference between the untreated mutant worms and the MeHg-treated mutant worms. Combining these data suggests that the effects of MeHg on lipid storage were not dependent on the expression of *mir-34* or *mir-355*. In contrast, untreated *mir-124* mutant worms had similar lipid storage levels to untreated N2 worms (Figure 6B). Additionally, lipid storage levels were elevated in *mir-124* mutant worms exposed to MeHg; however, they were significantly lower than N2 worms treated with MeHg (Figure 6B). This suggests that *mir-124* may be required for MeHg-induced lipid dysregulation.

In examining lipid storage in deletion mutants for the anti-adipogenic miRNA sequence strains, we expected the untreated mutants to have higher lipid storage levels than untreated N2 worms. Indeed, all three mutant strains had elevated lipid storage in untreated worms as compared to untreated N2 (Figure 6D–F). Mutant *mir-240* and the *mir-240* and *mir-786* worms treated with MeHg were not statistically different in terms of lipid storage level as compared to untreated mutant worms, nor were they different from the N2 worms treated with MeHg. These data suggest that neither MeHg-induced lipid dysregulation is independent of *mir-240* or *mir-786*. This was not the case for *let-7*. Untreated mutant *let-7* worms had significantly higher levels of lipid storage than the untreated N2 worms, and treatment with MeHg raised lipid storage levels significantly higher than the mutant *let-7* untreated worms or the N2 MeHg-treated worms (Figure 6D). This suggests that *let-7* may be an important regulator of MeHg-induced lipid dysregulation.

## 4. Discussion

MeHg is a neurotoxic agent that has recently been associated with metabolic effects. Due to evolutionarily conserved lipid metabolic pathways, *C. elegans* is a useful model organism to study the effects of metabolic changes induced by MeHg [44]. We have previously shown that MeHg increases levels of adipogenic transcription factors implicated in MS including *cepb-1* (ortholog to human C/EBP), *nhr-49* (ortholog to human peroxisome proliferator-activated receptor gamma, PPARγ), and *sbp-1* (ortholog to human sterol response element binding protein-1, SREBP-1), as well as a number of other genes involved in lipid synthesis and transport [17,18]. Additionally, we have shown that lipid dysregulation in response to MeHg exposure can be modulated with miRNA expression [19]. MiRNA are small non-protein coding RNA that silence gene expression by targeting specific mRNA sequences for degradation, thus preventing their translation. Deletion of miRNA processing machinery or the RNA polymerases that synthesize miRNA significantly altered toxicity, lipid accumulation, and oxidative stress in response to MeHg in worms [19]. While our initial studies focused on the effects of altering global miRNA expression, we were therefore interested in whether MeHg altered specific miRNA strains important for regulating lipid homeostasis. Herein, we demonstrate that MeHg alters the expression of specific miRNAs, leading to altered levels of their target mRNA sequences, and that the deletion of miRNA has functional consequences for lipid accumulation in worms.

It has previously been reported that MeHg decreases the expression of specific miRNA sequences in multiple models, including in immortalized human embryonic neural progenitor cells, cortical neurons, and in *C. elegans* [45,46,47]. Additionally, Hu et al. have found miRNA sequences that are up-regulated with MeHg exposure in zebrafish [48]. It is important to note that we currently do not know the mechanism of how miRNA expression is altered by MeHg. There are multiple possibilities, from altering miRNA gene expression to miRNA processing, miRNA stability, or gene silencing functions. Further research on this mechanism of action is essential. To date, it is unknown whether MeHg alters the expression of miRNAs that regulate lipid homeostasis. We selected six miRNA sequences in *C. elegans* that have homology to miRNA sequences that have been shown in mammalian systems to be either increased or decreased in the serum, adipocytes, or liver of individuals with MS. This is the first study to address whether these *C. elegans* sequences have similar effects on lipid metabolism as those that have been established in mammals.

*C. elegans*’ *mir-34* is highly conserved with human miR-34A, miR-34B, and miR-34C [27]. In diet-induced obese mice, miR-34a targets fatty acid metabolism and cholesterol biosynthesis in the liver by targeting the histone deacetylase SIRT1, which has inhibitory roles in these processes [49]. miR-34 is up-regulated in adipose tissue of obese mice and in 3T3-L1 adipocyte cells *in vitro*, and is involved in insulin resistance [50]. Additionally, miR-34 is overexpressed during differentiation processes involved in cytokine-mediated β-cell dysfunction in non-obese diabetic mice [51]. Known targets of miR-34 include SIRT1, uncoupling protein 1 (UCP1), PPARγ coactivator 1α (PGC-1α), and AMP-activated protein kinase (AMPK) [40,49,52]. *C. elegans*’ *mir-124* is highly conserved with human miR-124 [27]. In mammals, *miR-124* is involved in insulin release from β-cells, and has an adipogenic effect-targeting Dlx5 transcription factor that affects cell differentiation in 3T3-L1 adipocyte cells [53,54]. Furthermore, miR-124 is overexpressed in visceral adipose tissue of obese children [55]. Known targets of miR-124 include adipose triglyceride lipase (ATGL), preadipocyte factor 1 (Pref-1), and tribbles homolog 3 (TRB3, a modulator of AKT signaling [41,56,57]. Finally, *mir-355* has been shown to regulate insulin signaling by targeting the insulin-like growth factor 1 receptor (IGF1R) [39]. Conversely, *C. elegans*’ *mir-240* is highly conserved with human miR-193a and miR-193b [27]. In mammals, miR-193b regulates adiponectin production in white adipose tissue, as well as inducing myoblast differentiation into brown adipocytes [42]. Additionally, miR-193b is one of several miRNAs that gets down-regulated in high-fat diet-induced obesity in mice [58]. Known targets of miR-193b include AKT, cAMP-responsive element-binding protein (CREB), nuclear transcription factor γ (NF-γ), and adiponectin receptor [42,59]. *C. elegans*’ *mir-786* is highly conserved with human *miR-365* [27]. MiR-365 is an anti-adipogenic miRNA that clusters with miR-193 and regulates brown adipose tissue differentiation [43]. Known targets of *mir-365* include Runx1t1 runt-related transcription factor 1 (Runx1t1), UCP1, PGC-1α, PPARα, and PPARγ [43]. As previously mentioned, *let-7* is a multifunctional miRNA found to inhibit adipogenesis and regulate insulin resistance [30,31]. Known mRNA targets of *let-7* include PPARγ, FABP, and AMPK [30,60].

Overall, we found that expression of miRNA sequences in response to MeHg varied with the time post exposure. All six miRNA sequences were increased in expression 24 h post MeHg exposure as compared to untreated control worms. At 48 h post MeHg exposure, however, there were differences in expression between the adipogenic and anti-adipogenic miRNA sequences in the worms. At 48 h post MeHg treatment, anti-adipogenic miRNA sequences (*let-7*, *mir-240*, and *mir-786*) were decreased in response to MeHg and the adipogenic sequences *mir-124* and *mir-34* were increased in response to MeHg as compared to the untreated control worms. At this time, it is unclear why miRNA expression varied significantly post MeHg exposure. It is known that expression of miRNA is a dynamic process that fluctuates following developmental and environmental signals. Sun et al. have found that the expression of mmu-miR-15a-5p, mmu-miR-125b-5p, and mmu-miR-132–5p fluctuated in expression following ischemic transient common carotid artery occlusion in Mongolian gerbils (*Meriones unguiculatus*), leading to altered expression of target genes TDP43, FUS/TLS, and Hsp70 in the hippocampus [61]. Similar fluctuations in miRNA expression were found after 2 months post birth in mouse hippocampus following neonatal hypoxia [62]. Time-dependent changes in miRNA levels have also been observed for tumorigenesis, infection, and adipogenesis [63,64,65]. Therefore, it should not be surprising that the miRNA levels fluctuated post MeHg exposure as the worms developed from L1 stage to adulthood.

In the examination of both miRNA expression and mRNA expression post MeHg exposure, we found that certain miRNA sequence–mRNA target relationships were well conserved between humans and *C. elegans*. These included *mir-124* and *atgl-1*, *let-7* and *lbp-5*, as well as *mir-240* and *mir-786* with *paqr-2*. These relationships followed the expected directionality, i.e., decreased expression of *let-7* occurred with increased expression of its target gene *lbp-5*. Furthermore, the shape of the dose–response curves for the miRNA expression matched the shape of the dose–response curve for mRNA target gene expression, further strengthening the relationship between the two genes. These data suggests that functions of these miRNA are evolutionarily conserved. It is important to note that in our study, we focused only on one mRNA target per miRNA sequence. As there are multiple mRNA targets for each miRNA, it will be important to determine whether other known mRNA target genes are similarly regulated by *mir-124*, *let-7*, *mir-240*, and *mir-786* in *C. elegans*. Furthermore, since the miRNA–mRNA relationship was not confirmed by *mir-34* and *aak-2*, there is a possibility that other mRNA target genes may actually show the miRNA–target mRNA relationship in worms. Finally, our data suggest that the effects of MeHg on miRNA sequences in *C. elegans* may have functional consequences in mammalian systems.

Genetic strains lacking each of the miRNA strains evaluated in this study were used to determine whether the miRNA sequence plays an important role in lipid accumulation in worms in the presence or absence of MeHg. Worms that were deficient in *mir-34*, *mir-124*, *mir-355*, *let-7*, *mir-24*, or *mir-240* and *mir-786* all were more sensitive to MeHg than N2 worms; dose–response survival curves for all the mutant strains were shifted to the left of N2 worms. These data are in agreement with our previous findings that worms that are deficient in miRNA due to a mutation in either *pash-1* (partner of Drosha) or *nrde-2* (nuclear RNAi defective) are more sensitive to MeHg [19]. MiRNA have multiple roles in metabolism and stress responses, as well as in development. As both the deletion of adipogenic or anti-adipogenic miRNA sequences made the worms more sensitive to MeHg, it is hypothesized that their roles in survival are independent of their roles in lipid regulation. It is unclear at this time how deletion of these miRNA sequences cause the worms to be more sensitive to MeHg.

Due to the increased sensitivity of the miRNA mutant worms to MeHg, we used significantly lower concentrations of MeHg than we have previously used in N2 worms to investigate lipid accumulation. Herein, we show that concentrations of MeHg as low as 0.25 μM increased lipid storage in N2 worms as measured with Nile Red staining. It was expected that when an adipogenic miRNA sequence was mutated, MeHg-induced lipid accumulation would be attenuated. This finding was observed for *mir-124* mutant worms in response to MeHg, but not for *mir-34* or *mir-355* mutant worms. This suggests that *mir-124* induction in response to MeHg has functional consequences in lipid accumulation. Previous studies of *mir-124* in *C. elegans* has implicated the role of miRNA in neuronal functions such as neural cell specification, long-range patterning, and sensory neuronal morphogenesis [66,67,68]; however, its role in lipid metabolism had not been explored. Our data show a conserved role for *mir-124* in lipid accumulation in worms and mammals. We also expected that mutation of an anti-adipogenic miRNA sequence would augment MeHg-induced lipid accumulation. Mutation of *let-7*, *mir-240*, and *mir-240* and *mir-276* increased lipid accumulation independent of MeHg treatment, demonstrating their conserved role in regulating lipid accumulation. These data agree with previous reports of *let-7* regulating lipid levels in *C. elegans* [32]. Only mutation in *let-7* augmented lipid accumulation in response to MeHg, suggesting *let-7* down-regulation in response to MeHg has functional consequences.

## 5. Conclusions

Altogether, this study demonstrates conserved roles in lipid accumulation and mRNA gene expression in humans for four of the six investigated miRNA sequences in *C. elegans* (the adipogenic *mir-124* and the anti-adipogenic *mir-240*, *mir-276*, and *let-7*). Of these four sequences, only *mir-124* and *let-7* were responsive to MeHg treatment. Our study was limited to only six miRNA sequences and one target mRNA sequence for each miRNA. Since there are multiple mRNA targets per miRNA, there can be differential effects of MeHg on these sequences. Further profiling of miRNA and target mRNA sequences in response to MeHg exposure is warranted to have a better understanding of mechanisms of metabolic alterations of lipids by MeHg.

## Figures and Tables

**Table 1:** miRNA and target mRNA sequences.

miRNA	Effect	mRNA Target Gene (Mammalian Homolog)	References
*mir-355*	Adipogenic	*daf-2* (insulin-like growth factor receptor 1)	[39]
*mir-34*	Adipogenic	*aak-2* (AMP kinase)	[40]
*mir-124*	Adipogenic	*atgl-1* (adipose triglyceride lipase)	[41]
*mir-240*	Anti-adipogenic	*paqr-2* (adiponectin receptor)	[42]
*mir-786*	Anti-adipogenic	*paqr-2*	[43]
*let-7*	Anti-adipogenic	*lbp-5* (Fatty acid-binding protein 4)	[30]

**Table 2:** MeHg is more toxic in transgenic worms lacking specific miRNA sequences.

Strain	LD_50_ (μM)	*p*-Value
N2	20.43	
Mutant *mir-34*	2.53	<0.0001
Mutant *mir-124*	0.66	<0.0001
Mutant *mir-355*	1.43	<0.0001
Mutant *let-7*	3.95	<0.0001
Mutant *mir-240*	1.77	<0.0001
Mutant *mir-240* and *mir-786*	1.66	<0.0001

## Data Availability

The data presented in this study are available on request from the corresponding author.
